# Resistance Mechanism of Acute Myeloid Leukemia Cells Against Daunorubicin and Cytarabine: A Literature Review

**DOI:** 10.7759/cureus.33165

**Published:** 2022-12-31

**Authors:** Elly Y Arwanih, Melva Louisa, Ikhwan Rinaldi, Septelia I Wanandi

**Affiliations:** 1 Doctoral Program in Biomedical Sciences, Faculty of Medicine, Universitas Indonesia, Jakarta, IDN; 2 Pharmacology and Therapeutics, Faculty of Medicine, Universitas Indonesia, Jakarta, IDN; 3 Division of Hematology and Medical Oncology, Department of Internal Medicine, Cipto Mangunkusumo National General Hospital, Faculty of Medicine, Universitas Indonesia, Jakarta, IDN; 4 Biochemistry and Molecular Biology, Faculty of Medicine, Universitas Indonesia, Jakarta, IDN

**Keywords:** cytarabine, daunorubicin, acute myeloid leukemia, treatment, chemotherapy, resistance, leukemia, aml

## Abstract

Acute myeloid leukemia (AML) is a hematological malignancy commonly found in adult patients. Low overall survival and resistance to therapy are the main issues in AML. The first line of treatment for AML chemotherapy is the induction phase, namely, the phase to induce remission by administering a combination of daunorubicin (DNR) for three days followed by administration of cytarabine (Ara-C) with continuous infusion for seven days, which is referred to as "3 + 7." Such induction therapy has been the standard therapy for AML for the last four decades. This review article is made to discuss daunorubicin and cytarabine from their chemical structure, pharmacodynamics, pharmacokinetics, and mechanisms of resistance in AML.

## Introduction and background

Acute myeloid leukemia (AML) is a hematological malignancy characterized by blockade in the differentiation process of hematopoietic progenitor cells of myeloid series and rapid proliferation of immature myeloid cells, resulting in the accumulation of blast cells in the bone marrow [1]. The consequence of this process is the disruption of the formation of blood cells from the myeloid series, causing pancytopenia (thrombocytopenia, leukopenia, and anemia) [1].

Standard chemotherapy as the first line of treatment for AML is induction therapy by giving daunorubicin (DNR) or idarubicin (IDA) combined with cytosine arabinoside (cytarabine, Ara-C). Following induction therapy, patients are often given consolidation therapy, generally conducted by giving high dose cytarabine (100 mg/m^2^ and up to 3000 mg/m^2^ body surface area) or allogeneic hematopoietic stem cell transplant depending on cytogenetic and molecular classification [2]. Induction therapy is intended to induce remission while consolidation therapy is intended to remove any remaining leukemic cells. The average percentage of complete remission (CR) from induction chemotherapy in AML is 60-80% in young adults (<65 years) and 40-60% in old adults (>65 years) [3,4]. However, the relapse rate after induction therapy and consolidation therapy is still very high, namely, 50% in young adult patients (<60 years) and 80-90% in old adult patients (>60 years) [5].

It is known that therapy failure in AML patients is caused by drug resistance in leukemic stem cells [6,7]. Occurring drug resistance can be in the form of primary resistance and adaptive resistance (acquired resistance). Primary resistance occurs when the first treatment (induction) does not respond to the regimen given so that remission is not achieved, whereas adaptive resistance occurs if relapse occurs after the achievement of complete remission. Relapses can occur months or years after induction is complete [8]. In this article, we will discuss daunorubicin and cytarabine starting from their chemical structure, pharmacodynamics, pharmacokinetics, and their resistance mechanisms in AML.

## Review

When patients think about cancer therapy, the word they often think of is chemotherapy. Indeed, the general public associate cancer therapy with chemotherapy. While this is true even now, as the main treatment for most cancer remains chemotherapy, there are now other options such as immunotherapies and targeted therapies available for routine clinical use. However, most of the immunotherapies and targeted therapies are accessible only in developed countries due to the associated high costs of these treatments.

Many clinical guidelines also still recommend chemotherapies as the mainstay treatment with occasional combination with targeted therapies and/or immunotherapies based on molecular analysis of cancer. For AML, induction chemotherapy consisting of cytarabine and anthracycline is recommended by many clinical guidelines and the most used anthracycline is daunorubicin with a dose of 45 to 60 mg/m^2^ daily for three days [9,10]. The guideline from the National Comprehensive Cancer Network (NCCN) AML panel recommends cytarabine (100-200 mg/m^2^ continuous infusion) for seven days, together with either idarubicin (12 mg/m^2^ for three days) or daunorubicin (60-90 mg/m^2^ for three days) [9,11]. Guideline from European Leukemia Net (ELN) 2022 also stated intensive chemotherapy as the main treatment for AML [10]. Thus, knowledge of AML chemotherapy is important for clinicians treating AML patients and the mechanisms of chemotherapy resistance in AML are important to be discussed.

The chemical structure of daunorubicin and cytarabine

**Figure 1:**
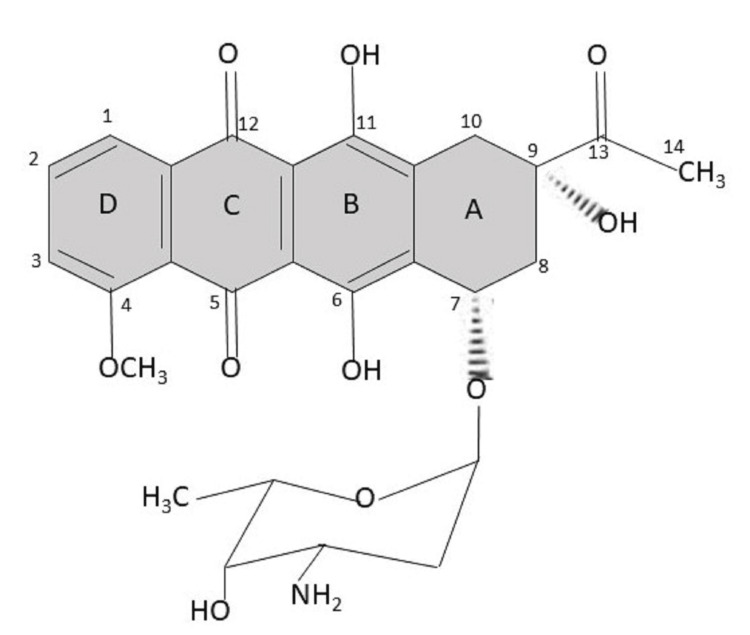
Daunorubicin structure Authors' creation.

Daunorubicin (DNR) is an antineoplastic agent of the anthracycline class of antibiotics, derived from the mutant isolate *Streptomyces peucetius* *var. caesius* [12]. These anthracycline class antibiotics include doxorubicin, idarubicin, and epirubicin [13]. Daunorubicin has the chemical formula C27H29NO10∙HCl, with a molecular weight of 563.99 and a pH of 4.5-6.5 in a solution of 5 mg/ml [6]. The structure of daunorubicin has aglycone and sugar groups (Figure 1). The aglycone group consists of a tetracyclic ring with quinone and hydroquinone groups on the C and B rings, a methoxy group on the D ring at the C-4 position, and a side chain on C-9 on the A ring with a carbonyl group on C-13. The sugar group called daunosamine is attached to C-7 of the A ring via a glycosidic bond and has an amine group at C-3 [14].

**Figure 2:**
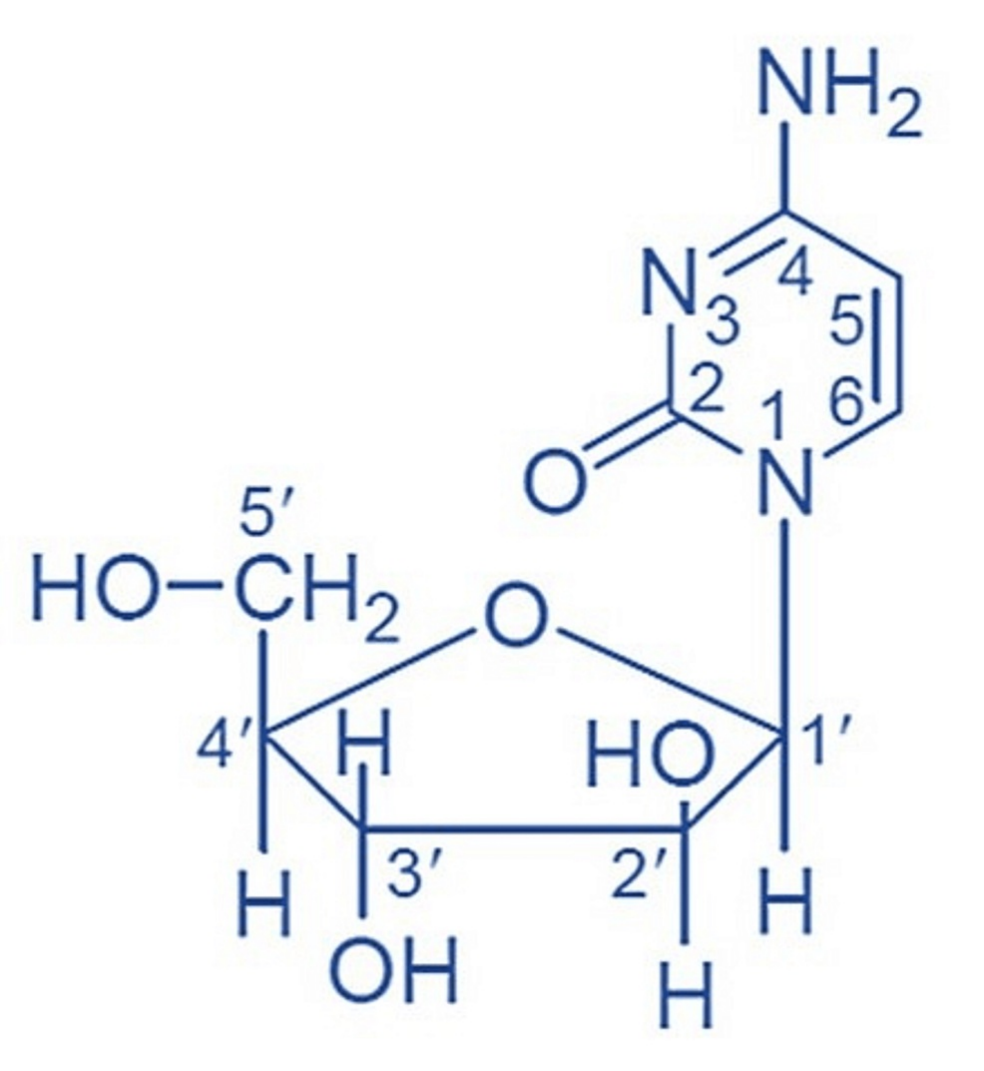
Cytarabine structure Authors' creation.

Cytarabine with another name cytosine arabinoside or Ara-C is an antineoplastic agent in the form of an analog of a pyrimidine nucleoside [15]. Cytarabine has other names, which are 1-beta-D-arabinofuranosylcytosine, 4-amino-1-beta-D-arabinofuranosyl-2(1H)-pyrimidinone, cytosine arabinoside, cytosine-1-beta-D-arabinofuranoside, and cytosine-β-D-arabinofuranoside, with the chemical formula C9H13N3O5 (Figure 2) and a molecular weight of 243.22. Cytarabine is in the form of powder and is soluble in liquid solvents (alcohol and chloroform) [6].

Pharmacodynamics and pharmacokinetics of daunorubicin

There are several mechanisms of daunorubicin as an antineoplastic agent. First, daunorubicin acts as a DNA intercalating agent [14,16]. The four A-D rings in daunorubicin can intercalate with DNA thereby inhibiting DNA replication and transcription. This causes the synthesis of DNA and RNA to be disrupted in the synthesis phase of the cell cycle. The second mechanism is by inhibition of topoisomerase II [17,18]. In the intercalation process, the position of the side chain at C-7 and C-9 of the A ring is located outside the DNA double strand. This side chain plays a role in the formation of the tripartite daunorubicin-DNA-topoisomerase II complex, which inhibits the reconnection of double strands of DNA by topoisomerase II in the process of DNA replication [14,16].

The third mechanism is the formation of free radicals by daunorubicin. Free radicals are formed from the reduction of the quinone group in daunorubicin by the cytochrome P450 reductase enzyme. The reduction of daunorubicin causes the addition of one electron. This electron quickly binds with oxygen to produce superoxide anion, which then produces hydrogen peroxide (H2O2). An increase in excess levels of reactive oxygen species (ROS) causes damage to important components in cells, causing cell death [14,19].

Daunorubicin is given by intravenous injection, but plasma levels of daunorubicin decrease rapidly due to tissue absorption. Thus, daunorubicin will be distributed in various organs of the body, especially in the kidneys, liver, lungs, and heart, then it will enter into cell components, especially nucleic acids. Additionally, it can pass through the bloodstream to the placenta [6]. Daunorubicin metabolism occurs in the liver and other tissues, mainly by the cytoplasmic enzyme aldo-keto reductase, producing daunorubisol, which is a metabolite with antineoplastic activity. Within 30 minutes, 40% of daunorubicin will be in the plasma and will increase to 60% within four hours. Daunorubicin has a half-life of 26.7 hours. Of the daunorubicin dose that enters the body, 25% will be excreted through urine and 40% through the excretion of bilirubin [6].

Pharmacodynamics and pharmacokinetics of cytarabine

**Figure 3:**
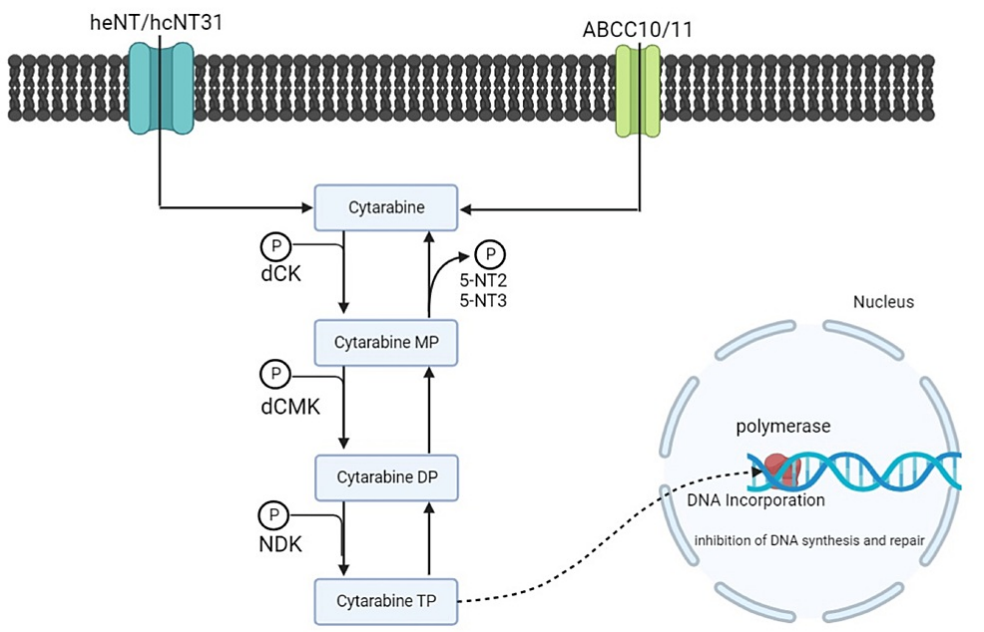
Mechanism of action of cytarabine as an anticancer drug dCK: deoxycytidine kinase; dCMK: deoxycytidine monophosphate kinase; NDK: nucleoside diphosphate kinase; hENT: human equilibrative nucleoside transporter; hCNT: human concentrative nucleoside transporter. Authors' creation.

Cytarabine enters cells through nucleoside transport proteins from the solute carrier (SLC) family, namely, human equilibrative nucleoside transporter (hENT) 1 and 2, and hCNT3 (human concentrative nucleoside transporter 3). In addition, cytarabine uptake is also regulated by the transmembrane proteins ABCC10 (MRP7) and ABCC11 (MRP8) (Figure 3) [15].

Cytarabine is then converted by the enzyme deoxycytidine kinase (DCK) into cytarabine monophosphate in cells. Furthermore, cytarabine is phosphorylated into cytarabine diphosphate and cytarabine triphosphate by the enzymes deoxycytidine monophosphate kinase (dCMK) and nucleoside diphosphate kinase (NDK). The form of cytarabine triphosphate (Ara-CTP) is an active form that can inhibit the action of DNA polymerase. Ara-CTP competes with deoxycytidine triphosphate (dCTP) in the process of DNA transcription. After cooperating with the DNA strand, Ara-CTP acts as a terminator chain thereby inhibiting DNA elongation, DNA synthesis, and DNA repair. The consequence of this is the blockade of the cell cycle from the G1 phase to the S phase, causing cell death [15,20].

After administration of cytarabine intravenously, distribution occurs in two phases: the first phase with a half-life of about 10 minutes, followed by a second phase with a half-life of one to three hours. After the distribution phase, more than 80% of the drug is in the plasma in an inactive form (1-βD-arabinofuranosyluracil). Within 24 hours, about 80% of the drug can be found in the urine in the non-toxic form of 1-βD-arabinofuranosyluracil (the inactive form of cytarabine) [6].

Role of gene mutations in AML chemotherapy resistance

The mechanism of AML cell resistance to chemotherapy can be caused by gene mutations. Among them are the FMS-like tyrosine kinase (FLT3) gene and DNA methyltransferase 3A (DNMT3A) gene mutations. The FLT3 gene is located on chromosome 13q12; it is a gene encoding the FLT3 tyrosine kinase receptor, which is expressed on the membrane of hematopoietic stem cells (CD34+) and other hematopoietic progenitor cells [21]. FLT3 is a class III receptor tyrosine kinase that can affect pathways such as the PI3K/Akt/mTOR, PI3K/Akt/bcl2-mcl2, RAS/MAPK/ERK, and JAK/STAT5/PIM1. These pathways are important in the regulation of cell proliferation, survival, and anti-apoptosis [22,23].

FLT3 receptor activation occurs due to the presence of FLT3 ligands, but mutations that occur in the FLT3 gene can cause FLT3 receptor activation without the presence of ligands, resulting in uncontrolled cell proliferation, survival, and anti-apoptosis. A common mutation in the FLT3 gene is internal tandem duplication (ITD), with base insertions varying from three to >1,000 bases in exons 14 and 15 in the juxtamembrane domain region [23]. The frequency of FLT3-ITD mutations in AML patients is known to be around 20-30% [6]. In a study conducted by Rinaldi et al., it was observed that the frequency of FLT3-ITD mutations in Indonesia is 21.5% in AML patients [24].

FLT3-ITD mutations in AML are known to be frequently found in patients with normal cytogenetics and are associated with a poor prognosis, especially in patients aged <60 years, with a low incidence of relapse (relapse) and overall survival (OS) [13,25]. In clinical studies conducted by Schnittger et al. and Kottaridis et al., it was found that FLT3-ITD mutations were associated with relapse and low OS [13,26].

FLT3-ITD mutations in leukemic cells have been shown to cause resistance to cytarabine [27,28]. Jin et al. stated that the K562 myeloid cell line with FLT3-ITD mutation has decreased expression of equilibrative nucleoside transporter-1 (ENT1) through increased expression of hypoxia-inducible factor 1 alpha (HIF-1α) [28]. ENT1 is known to be a transporter protein that plays a role in the uptake of cytarabine into cells, which means that a decrease in cytarabine uptake may be due to reduced ENT1 protein action [28]. FLT3-ITD mutations are also known to cause resistance to cytarabine through RUNX3 induction. Research conducted by Damdinsuren et al. showed that the knockdown of RUNX3 expression in the K562/FLT3-ITD cell line increased the sensitivity of cells to cytarabine administration [27].

According to several studies, DNMT3A is one of the most commonly mutated genes in AML [29,30]. However, the effect of this mutation is still debatable. A study by Yuan et al. showed that DNMT3A mutations caused poor response to aclarubicin in AML patients [31]. AML cells with DNMT3A mutation also have lower sensitivity to daunorubicin. A study by Chu et al. demonstrated that DNMT3A mutation activates nuclear factor-E2-related factor (NRF2) excessively, which then protects cells from apoptosis.

Both FLT3-ITD and DNMT3A are common mutations encountered in AML. FLT3-ITD is routinely checked in developed countries since it is used as risk stratification in clinical guidelines to determine treatments [9,10]. Other mutations that are commonly screened include IDH1, IDH2, and NPM1 [10]. Patients with FLT3-ITD mutation should be treated with FLT3 inhibitors as the prognosis with only chemotherapy treatment is poor. Currently, there are still many potential mutations being studied with the hope of finding potential targets for new drugs [32]. However, despite the promising era of targeted therapies as an add-on therapy, a chemotherapy regimen still remains the mainstay or backbone treatment of AML.

Mechanism of AML resistance to daunorubicin

The mechanism of AML resistance to daunorubicin can occur in several ways, namely, increasing the expression of the drug efflux transporter ATP-binding cassette (ABC), decreasing the activity of the target enzyme (DNA topoisomerase II), and the mechanism of resistance through the apoptotic pathway [33]. The drug efflux transporter protein that is often studied is P-glycoprotein, a member of the ABC family, which is encoded by the MDR1 gene and is also a multidrug resistance-associated protein (MRP). Overexpression of the two transporter proteins can cause AML cell resistance to the given anthracycline drug (daunorubicin) [34].

Stopping the activity of the DNA topoisomerase II enzyme is the target of therapy with a daunorubicin regimen. Therefore, reduced activity of the DNA topoisomerase II enzyme can cause AML cell resistance to daunorubicin. It was found that the activity of the DNA topoisomerase II enzyme was reduced in AML cells that were resistant to anthracyclines compared to AML cells that were sensitive to anthracyclines [35]. The mechanism of resistance of AML cells to daunorubicin is also known to occur through the apoptotic pathway due to mutations in the p53 gene as a tumor suppressor gene [36]. In adult AML cases, it was found that there was a p53 gene mutation in 15% of the total AML patient population [33].

Mechanism of AML resistance to cytarabine

A preliminary study of the pathways that play a role in the mechanism of transport, activation, and degradation of cytarabine in cells helps us understand the mechanism of resistance of AML cells to cytarabine. The first mechanism of resistance is caused by a deficiency of cellular uptake of cytarabine in cells [15,37]. This is caused by the reduced activity of nucleoside transporter proteins, especially the hENT1 transporter protein, which is caused by a mutation in the hENT1 protein-coding gene, the SLC29A1 gene [38]. In addition, the high activity of the ABCC10 and ABCC11 proteins causes a significant amount of cytarabine that has been in the cell to be pumped out of the cell in excess [39]. Reduced activity of DCK, CMPK, and NDK enzymes is also the cause of low phosphorylation activity, so cytarabine phosphorylation in cells decreases [15].

The second mechanism of AML cell resistance to cytarabine is the overexpression of enzymes that inactivate cytarabine such as cytidine deaminase (CDA), CMPD, and NT5C2 [15]. Meanwhile, the third mechanism is the domino effect of increasing dCTP in cells followed by overexpression of ribonucleotide reductase (RNR) or Ara-CTP and followed by an antagonistic effect of DNA incorporation of Ara-CTP. The final resistance mechanism is a change in DNA polymerase and an increase in the activity of DNA repair genes, such as the X-ray cross-complementing (XRCC) and excision repair cross-complementing (ERCC) groups [15].

The third mechanism is the formation of free radicals by daunorubicin. Free radicals are formed from the reduction of the quinone group in daunorubicin by the cytochrome P450 reductase enzyme. The reduction of daunorubicin causes the addition of one electron. This electron quickly binds with oxygen to produce a superoxide anion, which then produces H2O2. An increase in excess levels of ROS causes damage to important components in cells, causing cell death [14,19].

Clinical studies on chemotherapies

Currently, chemotherapies remain the main treatment of AML, especially in developing countries. Thus, many trials on chemotherapies are still ongoing such as trials involved in evaluating induction doses of daunorubicin or idarubicin. For example, the trial by Burnett et al. compared a daunorubicin dose of 90 mg/m^2^ versus 60 mg/m^2^ for AML induction [40]. The result of the trial showed that there was no overall difference in complete remission rate and two-year survival rate. Similarly, a study by Ohtake et al. also showed no difference between high-dose daunorubicin and standard-dose idarubicin [41].

It is actually recommended to combine chemotherapies with other treatments nowadays to prevent relapse from the minimal residual disease [42]. The combination of chemotherapy with sorafenib was analyzed in a randomized, placebo-controlled trial conducted by Serve et al. in the year 2013 [43]. Here, the trial assigned 201 patients to either sorafenib or placebo in between the chemotherapy cycles. However, the result showed no benefit from adding sorafenib in improving outcomes of OS and event-free survival. No other trials of adding sorafenib to AML treatment are currently being conducted.

In AML with an FLT3-negative profile, gemtuzumab ozogamicin (GO) is combined with standard chemotherapies. A meta-analysis by Hills et al. showed that the addition of GO increases OS [44]. However, the increase in OS was not seen in AML with high-risk cytogenetics. On the other hand, AML with positive FLT3 mutation is treated with a combination of chemotherapies and midostaurin, an FLT3 inhibitor [10]. Currently, many therapies are now selected based on the molecular profile of cancer. Thus, we should expect better outcomes in the future from combined chemotherapies and targeted therapy.

Relapsed and refractory AML (R/R AML) is a major issue today for clinicians. Up to 40% of AML patients are unable to achieve complete remission even with a combination of intensive chemotherapy and targeted therapies [45,46]. These R/R AMLs are treated with a salvage regimen or reinduction regimen. Widely used for salvage therapy is the combination of fludarabine with idarubicin (FLAG-Ida). A study by Westhus et al. showed that only 56% of the patients achieved complete remission with this chemotherapy protocol [47]. For a list of common salvage regimens, readers should refer to ELN 2022 AML guideline [10].

Improvement in complete remission outcome is needed for R/R AML patients. One of the pathways that can be done is by further research into new chemotherapies. A trial by Ravandi et al. in refractory/early relapsed AML patients aged ≥60 years old compared vosaroxin plus cytarabine (versus placebo plus cytarabine) in 711 patients [48]. Voreloxin is novel chemotherapy that acts by intercalating DNA and poisons topoisomerase II but has different pharmacological properties and chemical scaffold [48,49]. The result of the trial showed better survival in the group treated with vosaroxin plus cytarabine (median OS of 6.5 months versus 3.9 months) [48]. Further studies are needed to evaluate the effect of adding vosaroxin.

The addition of hypomethylating (HMA) therapy with FLAG-Ida can also be used for a salvage regimen. Decitabine combined with aclarubicin and cytarabine (DAC) showed better efficacy than DAC alone. In some studies [50-52], novel therapies addition has been used for the treatment of R/R AML such as venetoclax (B-cell lymphoma-2 (BCL-2) inhibitor), sorafenib (tyrosine kinase inhibitors), crenolanib, and nivolimumab, and many others are still being studied [53-56]. However, more data are needed to confirm the efficacy of these therapies. Currently, studies that compare different intensive salvage regimens are very few and patients that fail salvage regimens are offered allogeneic hematopoietic stem cell transplants.

## Conclusions

Chemotherapies whether used as a single therapy or in combination with other therapies remain the mainstay treatment for AML currently. However, AML cells have several mechanisms such as drug efflux transporter, enzyme inactivation, gene mutations, and alteration of signaling pathways that can cause resistance to chemotherapies. The most common gene mutations in AML include FLT3-ITD and DNMT3A mutations. Additional studies should be conducted to improve the effectiveness of chemotherapy from pharmacological and pharmacokinetic perspectives, as it may improve the medical outcomes of AML patients.
